# Vibration Control of Scanning Electron Microscopes with Experimental Approaches for Performance Enhancement

**DOI:** 10.3390/s20082277

**Published:** 2020-04-17

**Authors:** Yun-Ho Shin, Seok-Jun Moon, Yong-Ju Kim, Ki-Yong Oh

**Affiliations:** 1 Department of Safety Engineering, Chungbuk National University, Chungbuk 28644, Korea; shinyh77@cbnu.ac.kr; 2 Department of System Dynamics, Korea Institute of Machinery and Materials, Daejeon 305-343, Korea; sjmoon@kimm.re.kr; 3 COXEM Co., Ltd., Daejeon 34025, Korea; kimyj@coxem.com; 4 School of Energy System Engineering, Chung-Ang University, Seoul 06974, Korea

**Keywords:** vibration isolation, vibration control, vibration of scanning electron microscope

## Abstract

A vibration isolator embedded in precision equipment, such as a scanning electron microscope (SEM), wafer inspection equipment, and nanoimprint lithography equipment, play a critical role in achieving the maximum performance of the equipment during the fabrication of nano/micro-electro-mechanical systems. In this study, the factors that degrade the performance of SEM equipment with isolation devices are classified and discussed, and improvement measures are proposed from the viewpoints of the measured image patterns and vibrations in comparison with the relevant vibration criteria. In particular, this study quantifies the image patterns measured using SEMs, and the results are discussed along with the measured vibration. A guide for the selection of mounting equipment is presented by performing vibration analysis on the lower mount of the dual elastic mount configuration applied to the SEM, as well as the image patterns analyzed with that configuration. In addition, design modifications for the mount and its arrangement are suggested based on impact tests and numerical simulations.

## 1. Introduction

The fabrication processes of nano/micro-electro-mechanical systems (MEMSs) have been significantly enhanced with recent technological advancements that include the introduction of new fabrication processes [1,2], addressing new materials [3], and deploying precise equipment [4] that can provide extremely high performance and resolution. However, the fabrication processes must satisfy the stringent requirements for environmental vibration under operational conditions to ensure reliable performance [5]. Hence, a vibration isolator embedded in the precise equipment plays a critical role in the fabrication of nano/MEMSs. 

To provide optimal environments for the fabrication processes and mitigate the concerns involving vibrations during operation, generic vibration criteria have been suggested for the MEMS fabrication equipment [6,7,8]. These criteria account for the design of the building in which the equipment is installed, the traffic volume around the building, and the resident population, among other factors. It is important to establish these generic vibration criteria because the vibrations transmitted from the surrounding environment not only deteriorate the performance of the equipment but also increase the defect rate. Consequently, environmental vibrations significantly decrease the production rate and economic feasibility of the fabrication processes.

Based on the generic vibration criteria, various studies have been conducted to develop vibration isolation devices or equipment for the isolation of environmental vibrations transmitted to precise equipment. These studies are classified into two categories, i.e., passive-or active-type vibration isolators. 

Studies on passive vibration isolators focused on high fidelity modeling approaches and new design concepts. Specifically, Lee et al. [9,10] suggested an accurate nonlinear model for a pneumatic vibration isolator by employing an orifice model and a procedure to optimize the orifice specifications using transmissibility analysis. These studies improved the prediction accuracy of the vibration responses of a pneumatic spring. Xu et al. [11] examined the effect of a diaphragm used as an air-tight seal on the vibration isolation performance from the perspective of stiffness. This study increased the settling time for internal and external vibrations. Hence, the performance of vibration isolators in commercialized equipment [12,13,14] can be presented through the transmissibility or settling time without considering the equipment to provide optimal isolator performance.

Studies on active vibration isolators focused on addressing new types of actuating methods, deploying novel actuators for vibration isolators, or applying robust non-linear control theories to overcome the performance limitations of passive vibration isolators. Palomares et al. [15] proposed a new passive isolation concept using negative stiffness through kinematic design. Pneumatic actuator [16,17,18,19], voice coil actuator [20], electromagnetic actuator [21], and piezoelectric actuator [22] have been proposed to overcome the performance deterioration around the natural frequency due to the application of a passive mount, as well as to maintain a fast settling time for the internally induced force. Kerber et al. [16] improved the vibration isolation performance near the natural frequency by directly controlling the dynamic pressure of a pneumatic vibration isolator with a servo valve. Kato et al. [17] deployed a precise control method for an active pneumatic vibration isolator through model-based control of the pneumatic valve. Shin and Kim [18] and Chen et al. [19] applied robust non-linear control theories, i.e., time delay control and fussy sliding-mode control, respectively, to account for the non-linearity of pneumatic systems. In the case of an electromagnetic actuator [20,21], the performance of the passive vibration isolator was improved by adding it as a non-contact-type isolator in parallel. It was also suggested that a piezoelectric actuator [22] should be applied as an active part in a series arrangement. 

Although intensive studies have been conducted, they focused on maximizing the isolation performance. This objective typically reduces the transmissibility and settling time from the perspective of the vibration isolator. Hence, it is difficult to apply the methods proposed by previous studies to a specific target system, and the performance of the entire system to which the mount is applied is typically not fully examined. In other words, there is a lack of studies that focus on how the operational conditions of the equipment affect them. 

In this study, the factors that inhibit the performance of equipment, including a scanning electron microscope (SEM) and an isolation device, are classified and discussed, and improvement measures are proposed from the viewpoints of both measured image patterns and vibrations in regard to the relevant criteria. In particular, this study quantifies the image patterns measured with an SEM instrument. The results are discussed in terms of the measured vibration. The results of the systematic analysis of the measured vibration suggest that the image post-processing computer must be separated from the main body of the SEM, and the lower structure supporting the main body should also be reinforced. A guide for selecting the equipment mount is also presented based on the vibration analysis of the lower mount of the dual elastic mount configuration applied to the SEM and analysis of the image patterns measured with the equipment configuration. In addition, by examining the impact test results and simulations of the upper mount, i.e., the electron gun and the mount supporting it, of the dual elastic mount configuration, design modifications for the mount and its arrangement are also suggested. Based on the current design, a structural modification is proposed such that the natural frequency in the rotational direction appears after the natural frequency in the vertical direction.

## 2. Experiments

### 2.1. Vibration Measurement Conditions for the Environment and Equipment Operation

**Figure 1:**
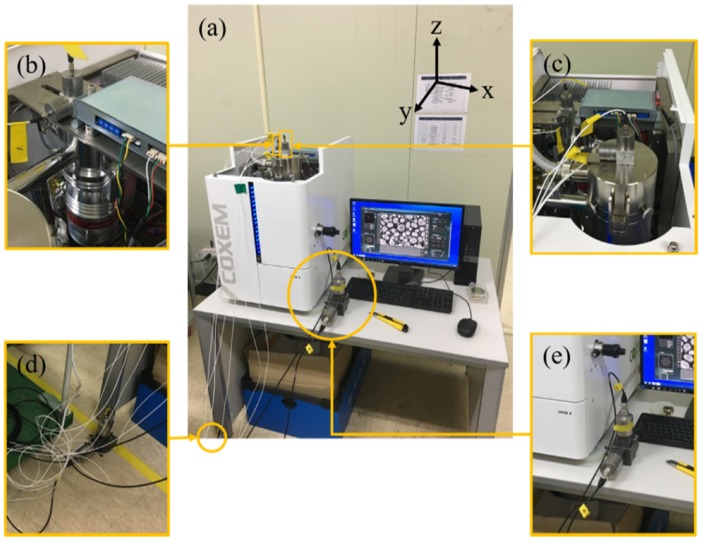
Overall configuration of the EM-30 equipment with accelerometers: (**a**) EM-30 consists of a main body, a computer for data acquisition and signal processing, and a table supporting the equipment; accelerometers were installed on (**b**) the pump with directions along the *y-* and *z-*axes, (**c**) electron gun with directions along the *x-* and *z-*axes, (**d**) floor (ground) with directions along the *y-* and *z-*axes, and (**e**) table with directions along the *y-* and *z-*axes.

This study considers an SEM instrument (EM-30, ×100,000 magnification, Tungsten filament type, COXEM, Daejeon, Korea) to optimize the vibration performance. Vibrations were measured using the general configuration of the EM-30 system (Figure 1a). The EM-30 system consists of a main body for mounting/scanning target samples, a computer for pre-/post-processing, and a table for loading. Eight accelerometers were installed to identify the vibration characteristics of each component. Specifically, two accelerometers with a sensitivity of 1 V/g were installed on the pump in the horizontal and vertical directions (*x-* and *z-*axes in Figure 1b) and on the electron gun in the horizontal and vertical directions (*y-* and *z-*axes in Figure 1c). Moreover, two accelerometers with a sensitivity of 10 V/g were installed on the floor and table in the horizontal and vertical directions (*x-* and *z-*axes in Figure 1d,e), respectively. Note that these accelerometers were installed to measure the floor vibration because accelerometers with higher sensitivity provide better performance for measuring nano- or micro-level vibrations; however, they can give rise to possible concerns of an added mass effect because accelerometers with high sensitivity have relatively large masses. Installing an accelerometer with a large mass in the target system can change the dynamic characteristics of the system of interest. This concern is negligible when measuring floor vibrations because the mass of the floor is significantly larger than that of the accelerometer. In contrast, accelerometers with a sensitivity of 1 V/g were installed on the SEM to measure the vibration of each element to mitigate concerns about altering the dynamic characteristics due to the accelerometer mass. The sensor selection process has been detailed in Appendix A.

### 2.2. Vibration Measurement

**Figure 2:**
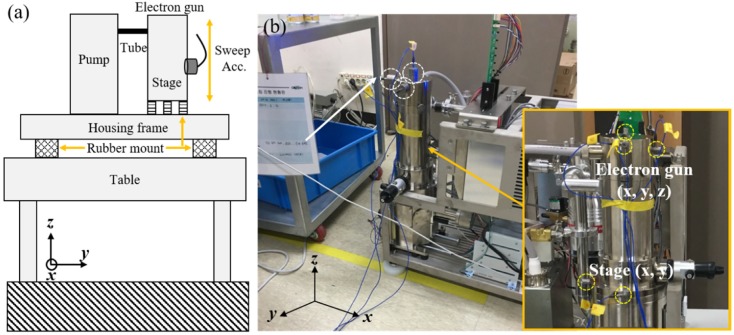
Experimental setup for vibration measurement: (**a**) schematic and (**b**) measurement location.

To elucidate the vibration characteristics of the SEM, vibration measurement was performed. The vibration measurement results provided a frequency response function for the system than can be used to identify its modes, i.e., natural frequencies. Figure 2a shows the schematic and measurement position of the experiment. Specifically, the SEM was uncovered and placed on the floor or a table to characterize the frequency response and coupled frequency response of the SEM with the table. Then, the system was excited to the frequency of interest using an impact hammer (086D05, PCB, Depew, NY, USA). The resulting vibration was measured with a trigger by sweeping accelerometers with a sensitivity of 100 mV/g from the stage where the sample was mounted to the top of the electron gun (Figure 2a); this method was used owing to a limited number of sensors. Vibration data were measured using Pulse (B&K, Denmark) with a sampling frequency of 400 Hz for a period of 8 s. A flat-top window was used for the measured impact force, whereas an exponential window was used for the measured vibration to eliminate leakage in the frequency domain [23]. Signal processing was performed to calculate the frequency response function, H(f), with a resolution of 0.125 Hz as follows.

(1) $$H\left(f\right)=\frac{S_{xy}\left(f\right)}{S_{xx}\left(f\right)},$$

where $S_{xy}\left(f\right)$ is the cross-spectral density function of the input signal x (the impulse force imposed) and output signal y (the measured acceleration), and $S_{xx}\left(f\right)$ is the autospectral density function of the input signal x (the impulse force imposed). The measured frequency response functions, H(f), were averaged five times to ensure that a reliable estimate of the structure transfer function was being guaranteed. The coherence function, $\gamma_{xy}^{2}$, was calculated to evaluate the consistency of the frequency response function obtained from the measurements, as well as to identify the excess noise or uncertainty between the input and output. The coherence function is calculated as follows.

(2) $$\gamma_{xy}^{2}=\frac{{\left|S_{xy}\left(f\right)\right|}^{2}}{S_{xx}\left(f\right)S_{yy}\left(f\right)},$$

where $S_{yy}\left(f\right)$ is the autospectral density function of the output signal y (the measured acceleration). Equation (2) yields a value between zero and unity. A value of unity at a given frequency indicates that all the response energy is caused by the stimulus or input signal, whereas a value of zero means that there is no correlation between the input force and output acceleration [24]. 

## 3. Results and Suggestions

This section consists of two sub-sections. The first sub-section analyzes the observed vibration characteristics. These analyses include the environmental vibration of the facility and the frequency response function of the system. Based on the experimental results, the second sub-section proposes a method of controlling the environmental/internal vibrations to improve the SEM performance. 

### 3.1. Characterization of Vibration

**Figure 3:**
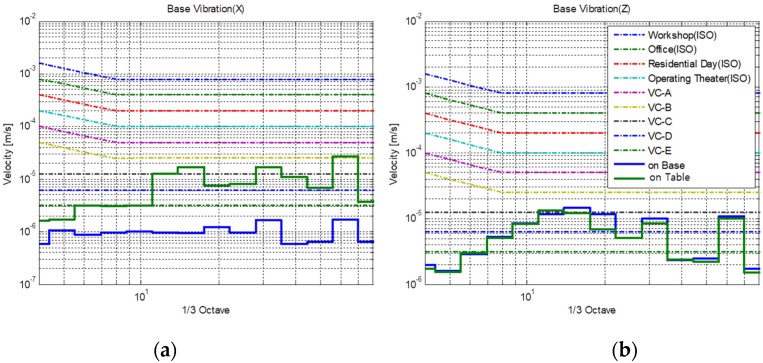
Vibration characteristics of the floor and table versus the frequency in the (**a**) horizontal (*x*-axis in Figure 1); (**b**) vertical (*z*-axis in Figure 1) directions.

Figure 3a,b present the vibration characteristics of the floor and table versus the frequency, along with the generic vibration criteria (VC, Appendix B) in the horizontal and vertical directions (*x-* and *z*-axes in Figure 1, respectively) during normal operational condition. The normal operational condition means that the SEM equipment was operated to measure the image pattern of a material of interest. More specifically, the electron gun measures a pattern, the vacuum pump runs at 90,000 rpm in the SEM equipment, and an air compressor of 60 Hz operates to supply air to the system. Vibrations on the floor and table in the vertical direction are smaller than the Class C VC curve throughout the region, except for certain frequencies (Figure 3b). Note that Class C is the vibration criteria for SEM and transmission electron microscopy (TEM) equipment. Even for the frequencies around 60 Hz where the vibration exceeds the criteria, the magnitude by which the criteria is exceeded is negligible. Hence, it can be inferred that vibrations in the vertical direction do not affect the performance of the SEM equipment based on the generic vibration criteria. In contrast, vibrations in the horizontal direction on the table exceed Class C at several frequencies and even exceed Class B at certain frequencies, whereas those in the horizontal direction on the floor satisfy the criteria for Class C as well as those for Class E. This observation suggests that vibrations in the horizontal direction are significant and deteriorate the performance of the SEM equipment. This is also evidenced by the blurred boundary of the pattern measured with the SEM, as illustrated in Section 3.2.1. 

There are two excitation sources in the horizontal direction on the table. One is the floor vibration, which can be amplified through the dynamic characteristics of the table. This vibration occurs over the entire interested frequency range, implying that the elastic modes that can cause unwanted table vibrations, other than the vibration isolation device installed in the equipment, should be designed to have the maximum stiffness outside the frequency range of interest. The other excitation source is operational vibration in the horizontal direction, which is transmitted from several components, including the pump and computer. The excitation from several components can also be amplified by the table owing to the table dynamics. Hence, the operational vibration from the equipment should be isolated from the table. In other words, in the SEM equipment set with the table, it is necessary to design the isolation element underneath the equipment to isolate the floor vibrations and modify the structural design, such as that of the table, that can amplify the vibrations from other components in the equipment or adversely affect the equipment. More details of the suggested methods to control the vibrations are discussed in the next sub-section.

**Figure 4:**
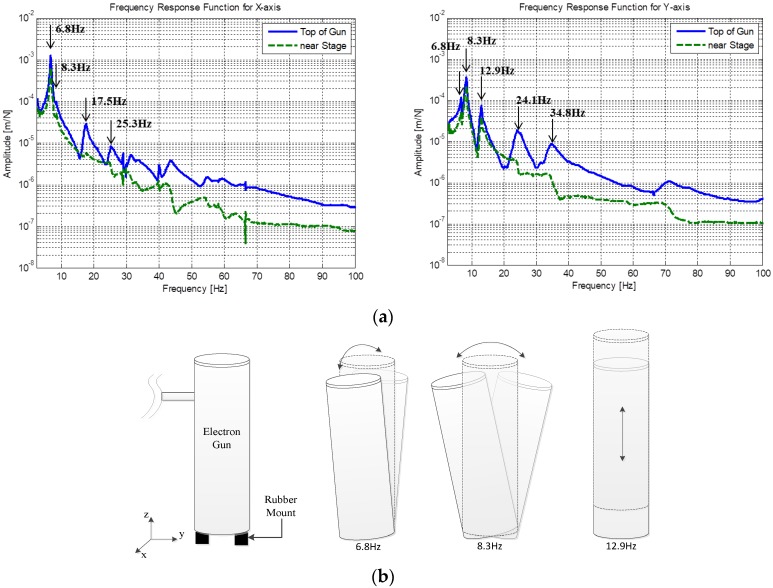
Vibration measurement under the normal operational condition. (**a**) Frequency response function; (**b**) Rigid body mode shapes of the electron gun.

An analysis of the vibration characteristics is presented in Figure 4a. The frequency response functions measured at two extreme locations are also presented. The blue line denotes the frequency response function measured at the top of the electron gun, whereas the green dashed line denotes the frequency response function measured at the location where the sample was mounted. The other frequency response functions are omitted for the sake of brevity because they exhibit similar trends with different magnitudes. It is evident from Figure 4a that the three dominant peaks that affect the performance of the equipment during operation are located in the low-frequency region. The first, second, and third rigid body modes are located at 6.8, 8.3, and 12.9 Hz, respectively, and their mode shapes are presented in Figure 4b. The mode shapes were identified based on the frequency response functions measured at several locations. The first mode is a *y*-axis rotational rigid body mode, the second is an *x*-axis rotational rigid body mode, and the third mode is a *z*-axis translational rigid body mode. This analysis of the mode shapes suggests that the first and second modes will adversely affect the performance of the equipment because these modes impede the alignment of the electron beam generated from the top of the electron gun with the sample placed under the electron gun. Therefore, it is preferable that the frequencies of the rotational modes are designed to be higher than those of other modes. A detailed improvement is suggested in the next sub-section. 

### 3.2. Design Suggestions

#### 3.2.1. Environmental Vibration Control

**Figure 5:**
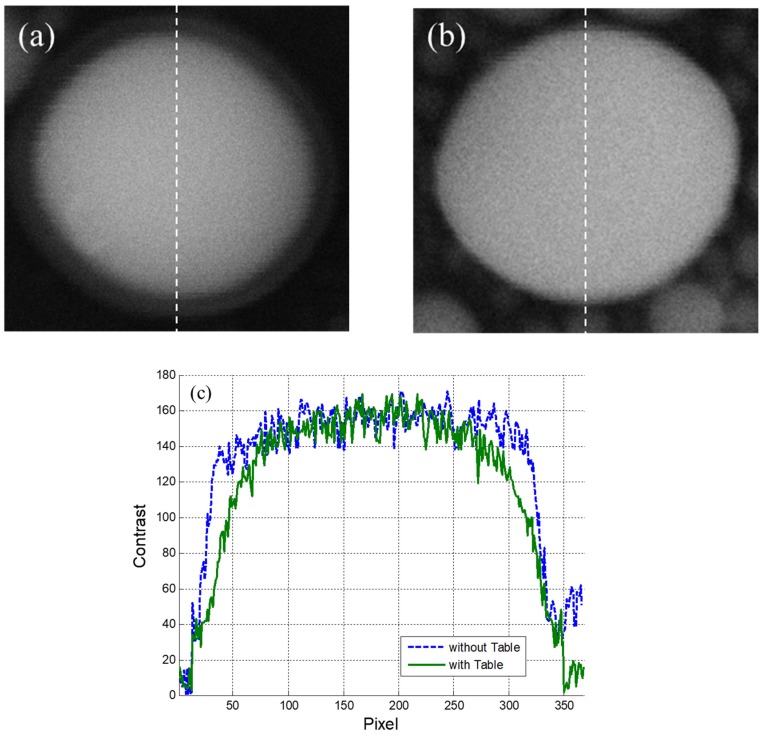
Measurement results for specimen patterns obtained (**a**) with and (**b**) without a table; (**c**) contrast variation along the specimen pattern images.

The patterns of a sample specimen measured with the SEM were compared to elucidate the problems due to the presence of the table (Figure 5). Figure 5a,b present the patterns of the sample measured with and without the table, respectively. These two figures clearly demonstrate that vibrations exceeding the criteria in the horizontal direction resulted in blurring of the measured image; thus, the SEM cannot ensure sufficient performance. This phenomenon was quantified through the contrast variations in the center of the images, which were measured following the dashed lines in Figure 5a,b and presented in Figure 5c. The solid line in Figure 5c denotes the contrast variation obtained with the table, whereas the dashed line describes that obtained without the table. This comparison indicates that the slope for the image measured with the table is more moderate than that measured without the table as a result of image blurring. Quantitatively, the upward slopes with and without the table were measured to be 3.0 and 5.3 contrast/pixel, respectively, whereas the downward slopes were −2.0 and −4.3 contrast/pixel, respectively. On average, the images measured without the table exhibited a 1.9 times more significant contrast variation compared to that measured with the table, thereby further suggesting that the environmental vibrations exceeding the criteria play a critical role in determining the equipment performance. 

**Figure 6:**
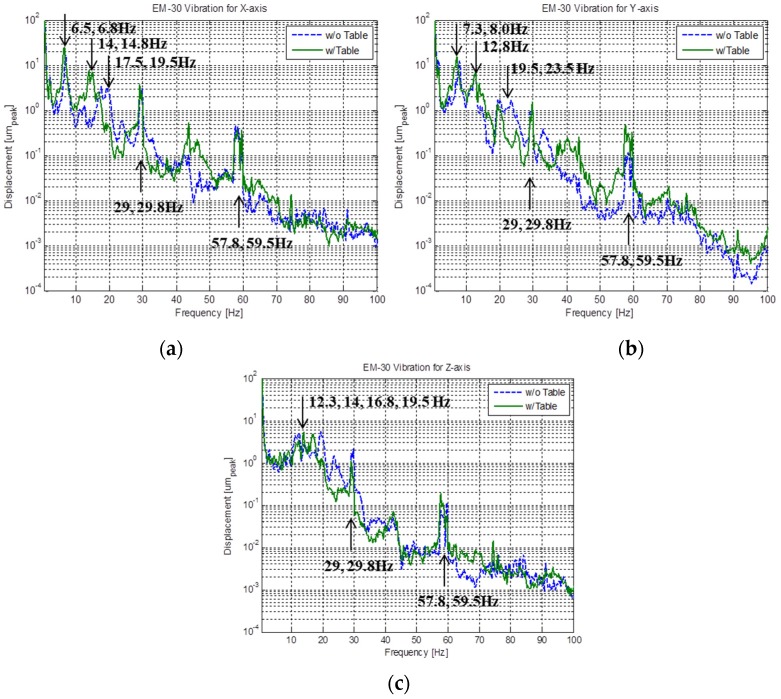
Frequency response functions obtained from vibration measurement in the directions of (**a**) x-axis; (**b**) y-axis; and (**c**) z-axis.

To examine the harmful influence of the dynamic characteristics of the table on the operation of the SEM quantitatively, frequency response functions for three directions were analyzed (Figure 6). The frequency of most modes decreases when a table is used, implying that placing the SEM on the table increases the equivalent mass or decreases the equivalent stiffness of the overall system. It should be noted that a peak between 57 and 60 Hz is significantly magnified. This peak is a high harmonic, i.e., the ninth harmonic, of the first mode, indicating that this mode overlaps with the operational frequency of the post-processing computer, and thus, magnifies the vibrations. This observation also corresponds to the mode shape analysis; the first mode is a rotational rigid body mode in the direction of the *y*-axis. It is inferred that the first mode and its high harmonics magnify in the horizontal vibrations, thereby exceeding the criteria. Moreover, vibrations in the horizontal direction are more important than those in the vertical direction from the design perspective of the SEM. The electron gun hangs from the body of the instrument like an inverted pendulum, suggesting that rotational motion will play a critical role in the performance of the equipment. In conclusion, vibrations in the horizontal direction originating from the computer are magnified by the table and act as an excitation force for the SEM, thereby resulting in the image measured by the SEM being blurred.

**Figure 7:**
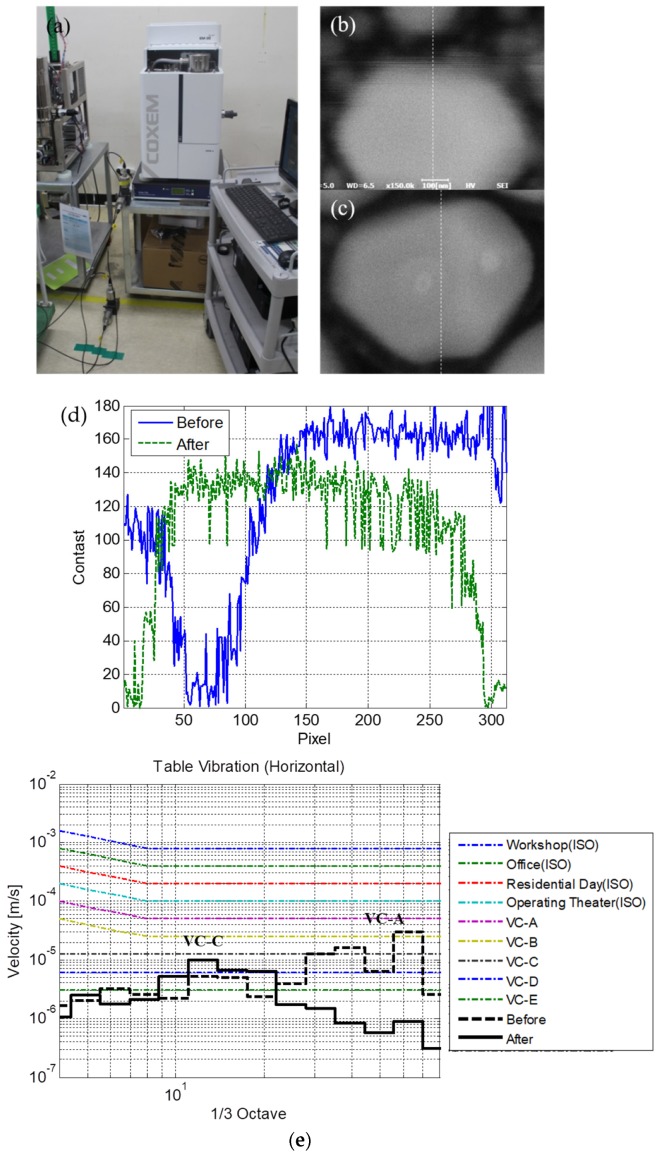
(**a**) Modified configuration of the overall system; measurement results for a specimen obtained using (**b**) wood and (**c**) steel table; (**d**) contrast variations in the specimen pattern images; (**e**) vibration levels compared to the criteria.

**Figure 8:**
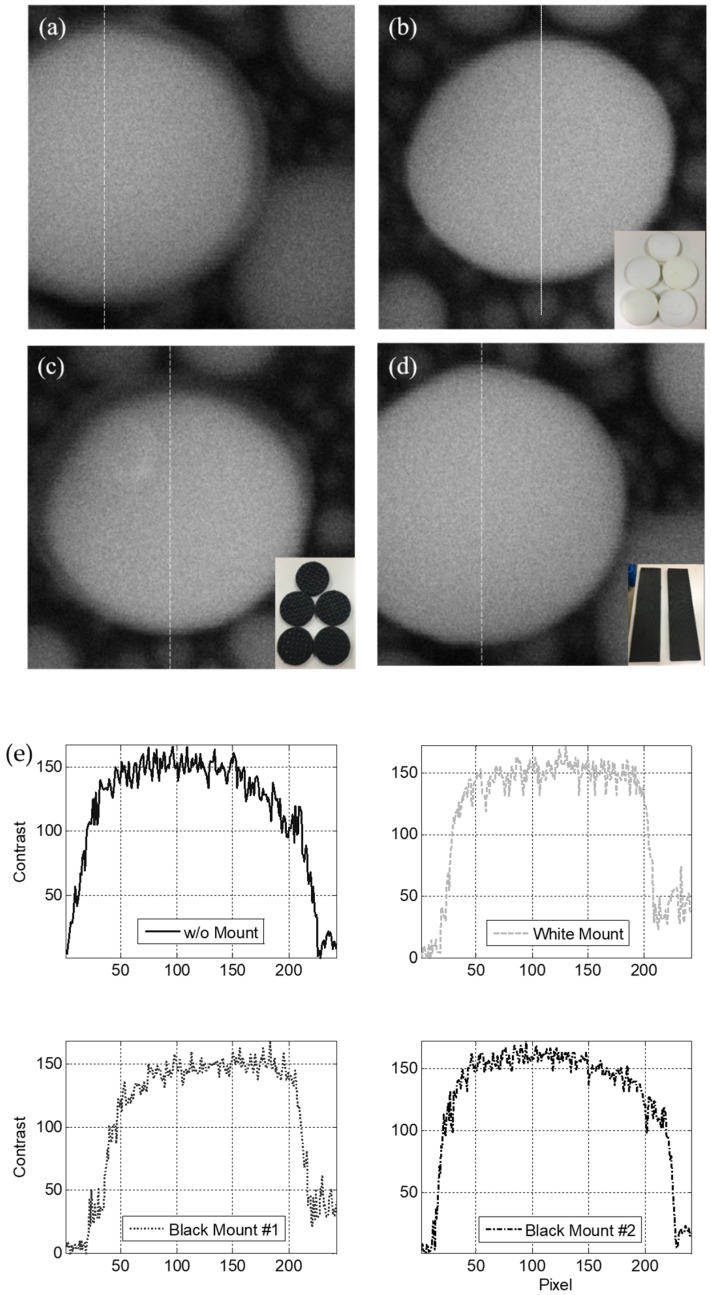
Specimen pattern images measured with (**a**) no mount, (**b**) white mount, (**c**) black mount #1, and (**d**) black mount #2 under operational vibration conditions; (**e**) contrast variations in the specimen pattern images.

Several methods have been proposed to control the vibrations of the table in the horizontal direction. In principle, a vibration source, i.e., the post-processing computer, should be isolated from the equipment. Moreover, the rigidity or equivalent stiffness of the table should be increased by adding a stiffness bar (or a reinforcing frame) or changing the material of the table. This study addresses both the methods. As illustrated in Figure 7a, the SEM was separated from the post-processing computer by placing both on two separate tables. Moreover, a steel frame table with a relatively high rigidity was used for the SEM. This modification improved the performance of the SEM. Specifically, the image measured with the steel table (Figure 7c) was less blurred in comparison with the image measured with the wood table (Figure 7b). The slope of the contrast variation increased from 2.6 contrast/pixel with the wood table to 4.3 contrast/pixel with the steel table. The slopes were computed from the contrast values of the white dotted lines in Figure 7b,c, and the contrast values according to the pixels of the dotted line are presented in Figure 7d. The vibrations in the horizontal direction corresponded to VC-A with the wood table and VC-C with the steel table, suggesting that the vibrations on the table significantly decreased with the implemented design modification, as illustrated in Figure 8e.

Another method of controlling the vibration characteristics of the SEM is deploying passive viscoelastic mounts on the housing frame of the SEM (Figure 2a). This study used three types of rubber mounts with different stiffnesses on the SEM, as illustrated in the insets of Figure 8b–d. The stiffness of black mount #1 was the lowest (inset of Figure 8c), followed by that of the white mount in the middle (inset of Figure 8b), whereas that of black mount #2 was the highest (inset of Figure 8d). In these experiments, the SEM was placed on the floor to observe the pure vibration characteristics of the SEM without the table. Then, the SEM performance was measured under normal operational and extreme environmental vibration conditions. The vibrations were controlled by preventing any personal movements for the normal operational vibration condition, whereas they were artificially controlled through personal movements for the extreme environmental vibration condition.

**Figure 9:**
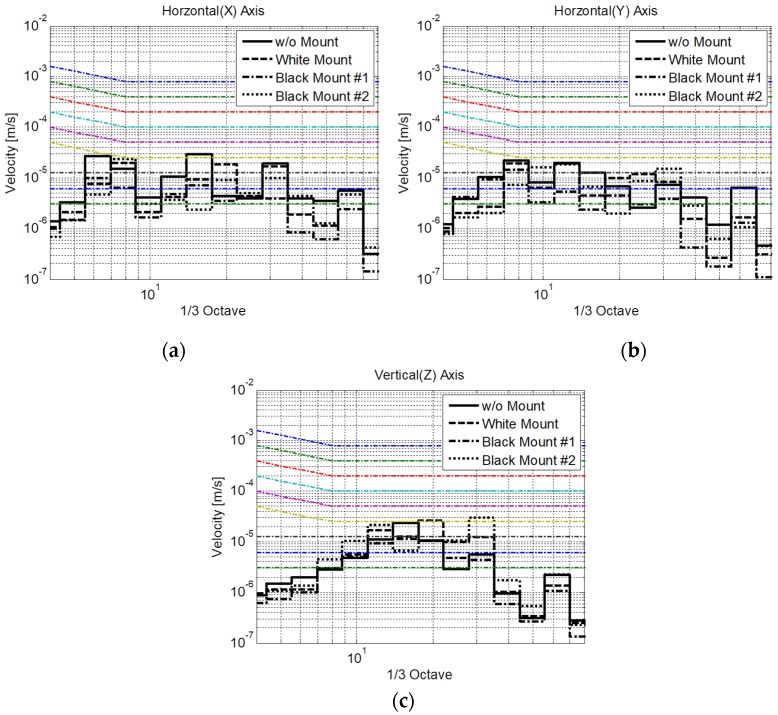
Operational vibration in the directions of (**a**) *x*-axis, (**b**) *y*-axis, and (**c**) *z*-axis compared to the generic vibration criteria with three different types of mounts.

When the system was exposed to normal operational vibration, the images obtained with all the types of mounts were less blurred (Figure 8b–d) in comparison with those measured without any mounts (Figure 8a). Quantitatively, the upward, downward, and mean slopes were found to be 4.2, −2.3, and 3.3 contrast/pixel, respectively, without mounts, as illustrated in Figure 8e. In contrast, the upward, downward, and mean slopes were 5.5, −9.0, and 7.3 contrast/pixel, respectively, with the white mount; 4.7, −7.7, and 6.2 contrast/pixel, respectively, with black mount #1; and 6.4, −10.1, and 8.3 contrast/pixel, respectively, with black mount #2. All mounts increased the slope of the contrast variation by at least a factor of two. The measured vibration was transformed to a velocity to allow for quantitative comparison with the generic criteria (Figure 9). The vibration levels corresponded to VC-A, VC-B, and VC-B without mounts in the directions of *x-*, *y-*, and *z-*axes, respectively. In contrast, the vibrations corresponded to VC-B, VC-B, and VC-A with the white mounts; VC-C, VC-B, and VC-C with black mount #1; and VC-B, VC-B, and VC-A with black mount #2 in the directions of the *x-*, *y-*, and *z-*axes, respectively (Table 1). Overall, the black mount #1 exhibited the best performance, suggesting that the use of cheap passive viscoelastic mounts on the housing frame can improve the vibration isolation performance of the precise equipment if viscoelastic mounts of proper stiffness are selected.

**Figure 10:**
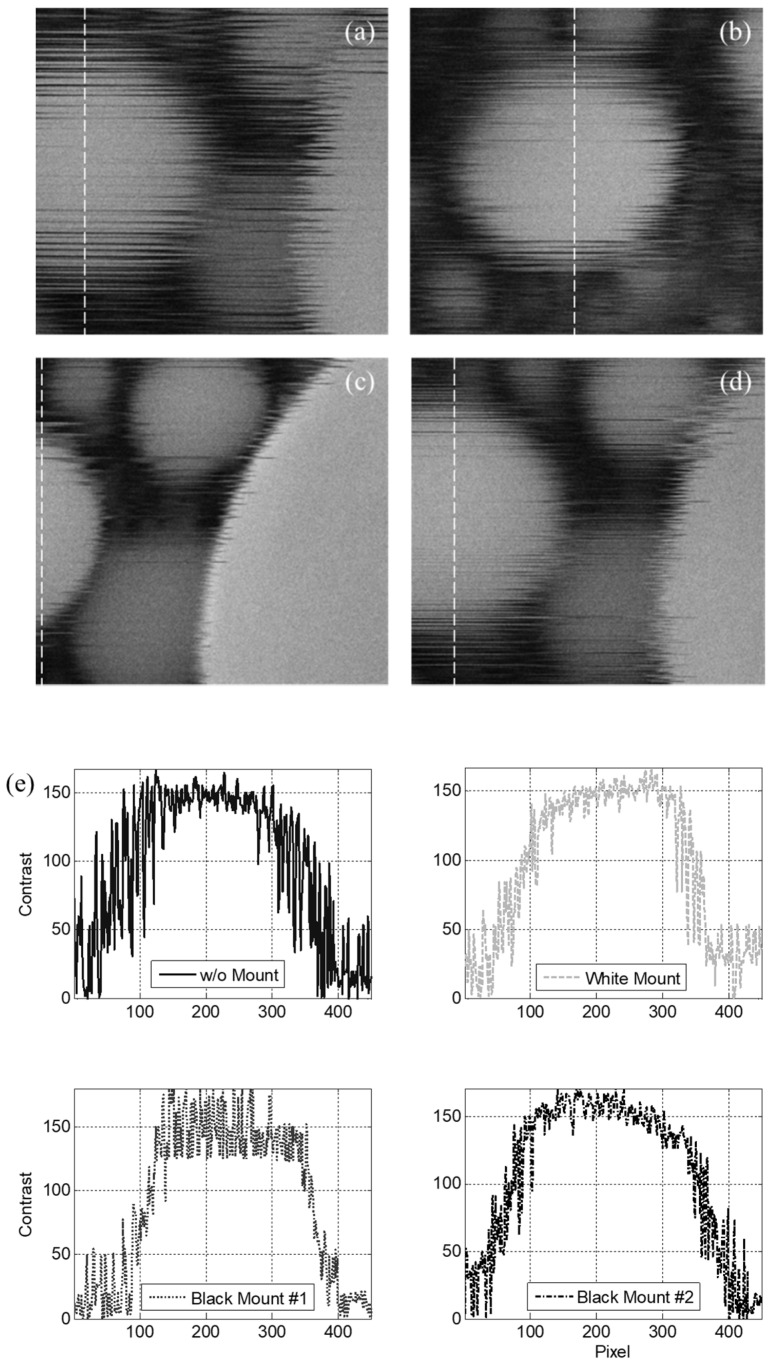
Specimen pattern images measured with (**a**) no mount, (**b**) white mount, (**c**) black mount #1, and (**d**) black mount #2 under extreme environmental vibration; (**e**) contrast variations in the specimen pattern images.

When the system was exposed to extreme environmental vibration, the images obtained with all the types of mounts were less blurred (Figure 10b–d) than those measured without mounts (Figure 10a). This comparison indicates that the softest mount had better performance than the stiffer mounts. This can be explained by the fact that the softest mounts isolate the SEM from the environment more effectively, particularly for high levels of floor vibration. Quantitatively, the upward, downward, and mean slopes were found to be 0.9, −0.9, and 0.9 contrast/pixel, respectively, without mounts, as presented in Figure 10e. In contrast, the upward, downward, and mean slopes were 1.4, −1.7, and 1.6 contrast/pixel with the white mount; 1.8, −1.7, and 1.8 contrast/pixel with black mount #1; and 1.7, −1.4, and 1.5 contrast/pixel with black mount #2, respectively. 

**Figure 11:**
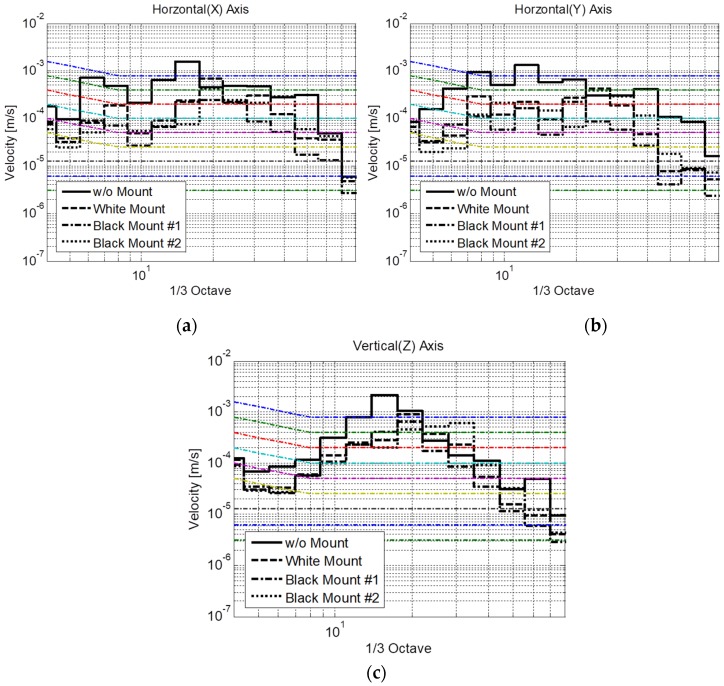
Environmental vibration in the directions of (**a**) *x-*, (**b**) *y-*, and (**c**) *z*-axes compared to the generic vibration criteria with three different types of mounts.

The extreme vibration was also compared to the generic vibration criteria (Figure 11). Without a mount, the vibration levels corresponded to the “Workshop” level for all directions. In contrast, the vibration level decreased to the “Office” or “Residential” level with the use of different mounts, as described in Table 1. Similar to the results under operational conditions, the vibration level with black mount #2 exhibited the best performance. From the analysis of two vibration sources, the softest viscoelastic mount, i.e., black mount #1, is suggested as the preferred mount for installation on the housing frame of the SEM. 

#### 3.2.2. Internal Vibration Control

**Figure 12:**
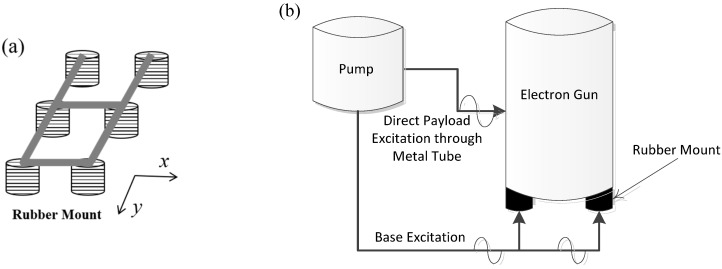
Schematic of the original six mounts supporting the electron gun.(**a**) Configuration of rubber mounts; (**b**) Schematic of electron gun system.

To ensure the SEM performance, it is necessary to control the internal vibrations transmitted from the pump, along with the environmental vibrations. The pump was used to maintain a vacuum at the location below the electron gun where the sample was mounted, as illustrated in Figure 2a. This pump is an internal source of cyclic vibrations (90,000 rpm). To isolate the vibrations transmitted from this pump, six elastic mounts were originally installed at the bottom of the electron gun, as illustrated in Figure 12a. It is necessary to modify the design of the isolation mounts installed under the electron gun based on the natural frequency of the modes and mode shapes, which are sensitive to the equipment performance. In designing the isolation element, it is better to reduce the stiffness of the isolator to eliminate the vibrations induced by the base excitation; however, the vibrations caused by the pump excitation force to maintain the vacuum act directly on the electron gun, thereby adversely affecting the SEM performance. Accordingly, it is necessary to improve the design by improving the isolation performance in the vertical direction and increasing the rigidity in the horizontal and rotational directions in which the pump vibration force acts. Figure 12b shows the external force acting on the electron gun, and Figure 12a shows the mounts at the bottom of the electron gun. To reduce the effect of rotational motion induced by the internal vibration source and sustain the isolation performance for the base excitation, it is desirable to increase the mode related to the rotational direction of the SEM while decreasing that related to the vertical direction. 

As a design improvement, lowering the translational rigid body mode in the direction of the *z*-axis (the third mode originally) is proposed with the objective that this mode will then become the first mode. In other words, the natural frequencies of the original first and second modes should appear after that of the mode related to the translational rigid body mode in the direction of the *z*-axis. This suggestion is made because the contribution of the first mode is greater than those of the other modes. 

**Figure 13:**
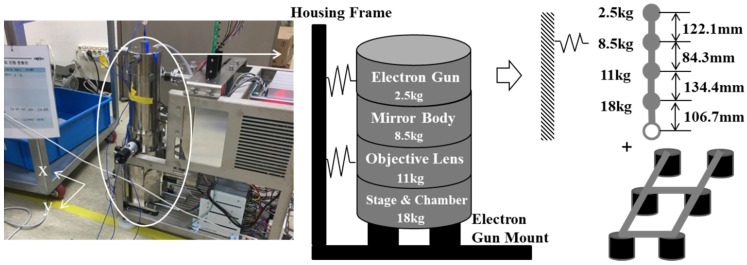
Simplified mathematical model of the electron gun–rubber mount system in the scanning electron microscope (SEM).

To predict the vibration performance of the proposed method, a three-degree-of-freedom model was built, assuming the electron gun to be a rigid body (Figure 13), as follows

(3) $$\left[\begin{array}{ccc} M & 0 & 0\\ 0 & I_{xx} & 0\\ 0 & 0 & I_{yy} \end{array}\right]\left[\begin{array}{c} \ddot{z}\\ {\ddot{\theta }}_{x}\\ {\ddot{\theta }}_{y} \end{array}\right]+\left[\begin{array}{ccc} c_{v,\;rubber} & 0 & 0\\ 0 & \begin{array}{c} c_{\theta_{x},\;rubber}\\ +c_{\theta_{x},\;tube} \end{array} & 0\\ 0 & 0 & \begin{array}{c} c_{\theta_{y},\;rubber}\\ +c_{\theta_{y},\;tube} \end{array} \end{array}\right]\left[\begin{array}{c} \dot{z}\\ {\dot{\theta }}_{x}\\ {\dot{\theta }}_{y} \end{array}\right]+\left[\begin{array}{ccc} k_{v,\;rubber} & 0 & 0\\ 0 & \begin{array}{c} k_{\theta_{x},\;rubber}\\ +k_{\theta_{x},\;tube} \end{array} & 0\\ 0 & 0 & \begin{array}{c} k_{\theta_{y},\;rubber}\\ +k_{\theta_{y},\;tube} \end{array} \end{array}\right]\left[\begin{array}{c} z\\ \theta_{x}\\ \theta_{y} \end{array}\right]=0,$$

where z, $\theta_{x}$ and $\theta_{y}$ denote the translational motion in the *z*-direction, rotational motion in the *x*-direction, and rotational motion in the *y*-direction, respectively; M, $I_{xx}$, and $I_{yy}$ denote the equivalent mass and equivalent moment of inertia in the direction of the *x*-axis and the equivalent moment of inertia in the direction of the *y*-axis, respectively. Note that this approach is effective for the system of interest where the three modes of interest are related to these motions. 

The equivalent mass, stiffness, and damping obtained in the impact tests for the target system are summarized in Table 2. The equivalent stiffness of the rubber mounts, $k_{v,\;rubber}$, was extracted from the natural frequency in the vertical direction, whereas the natural frequencies in the rotational direction were calculated using the equivalent mass and mass center of gravity, as illustrated in Figure 13. The difference between the calculated rotational natural frequencies and measured results was compensated by the equivalent stiffness from the tube connected to the electron gun. The vibration in the housing frame, i.e., the input to the gun, was measured.

**Figure 14:**
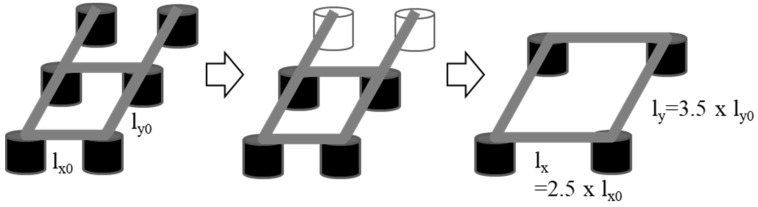
Suggestion for improved mount configuration design.

**Figure 15:**
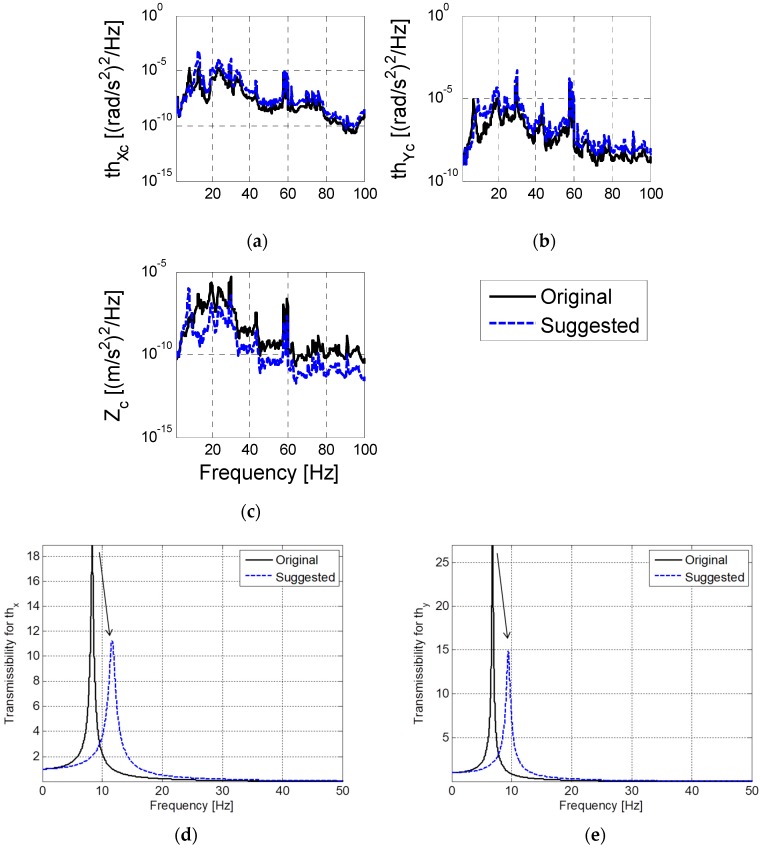
Performance estimation with the suggested mount configuration: estimated vibration at the center of gravity (CG) (**a**) in rotational x-axis($\theta_{x}$); (**b**) in rotational y-axis($\theta_{y}$); (**c**) in translational z-axis(z) and transmissibility in the rotational direction; (**d**) $\theta_{x}$; (**e**) $\theta_{y}$.

Based on the numerical simulations of the model and housing frame vibrations, decreasing the number of isolation devices from six to four and widening their placement positions are proposed, as illustrated in Figure 14. These changes are recommended to reduce the maximum amplitude and settling time in the rotational direction while lowering the natural frequency in the vertical direction. In addition, the elastic modulus of the viscoelastic material must be reduced by 50% to further lower the natural frequency of the vibration, as indicated in Table 3. By implementing these changes, the maximum amplitude of the transmissibility can be reduced, and the rapid attenuation of the residual vibration can be predicted when the electron gun is excited, as illustrated in Figure 15. This implies that the stiffness of the isolation element along the vertical direction must be designed to ensure a reduction in the overall vibration for the interested frequency range. Additionally, for horizontal vibrations that are sensitive to maximum displacement, a design is suggested in which the location of natural frequency is modified to be higher to ensure fast attenuation and small amplitude.

## 4. Conclusions

The conclusions drawn from this study can be summarized as follows.

- The configuration of the measuring instruments and the selection of sensors are discussed to measure the extremely small vibrations in an SEM and its environment, and an improved arrangement of the general configuration of the SEM equipment is proposed.
- An attempt is made to quantify the image patterns measured with the SEM; the post-processing computer and the main body of the SEM are separated, and the lower structure supporting the main body of the SEM are reinforced to reduce vibrations in this study. A mount selection guide for the dual elastic mount configuration is also presented. The proposed configuration is obtained by analyzing the measured image patterns and vibrations of the lower mount on the SEM.
- Design changes and mounting placements are proposed based on a review of the results of impact tests and simulations performed on the top mounted electron gun and mounting supports for the dual elastic mounting configuration. Based on the current design, structural changes are proposed such that the natural frequency in the direction of rotation will occur after the natural frequency in the vertical direction.

## Figures and Tables

**Table 1:** Vibration characteristics for three different types of mounts.

	Normal Operational Vibration			Extreme Environmental Vibration		
	x-axis	y-axis	z-axis	x-axis	y-axis	z-axis
w/o mount	VC-A	VC-B	VC-B	Workshop	Workshop	Workshop
White (9.8 × 10^5^ N/m)	VC-B	VC-B	VC-A	Office	Office	Workshop
Black #1 (8.8 × 10^5^ N/m)	VC-C	VC-B	VC-C	Residential	Residential	Office
Black #2 (10.1 × 10^5^ N/m)	VC-B	VC-B	VC-A	Office	Residential	Office

**Table 2:** Parameters used in the simplified mathematical model of the electron gun–rubber mount system.

Electron Gun			Rubber Mount		Tube	
			Stiffness	Damping	Stiffness	Damping
Mass, M	40 kg	Vertical Direction, (k_v,_ c_v_)	4.38 × 10^4^ N/m	62.9 N·s/m	-	-
Moment of Inertia, I_xx_	0.65 kgm^2^	X-Rotational Direction, (k_ϴx,_ c_ϴx_)	1.58 × 10^3^ Nm/rad	2.3 Nm·s/rad	1.89 × 10^2^ Nm/rad	0.1 Nm·s/rad
Moment of Inertia, I_yy_	0.65 kgm^2^	Y-Rotational Direction, (k_ϴy,_ c_ϴy_)	0.84 × 10^3^ Nm/rad	1.2 Nm·s/rad	3.44 × 10^2^ Nm/rad	0.1 Nm·s/rad

**Table 3:** Comparison of resonance frequencies between the original and suggested models.

	Original		Suggested	
	Resonance	Rank	Resonance	Rank
ϴy	6.8 Hz	1	9.4 Hz	2
ϴx	8.3 Hz	2	11.6 Hz	3
z	12.9 Hz	3	7.4 Hz	1

**Figure A1:**
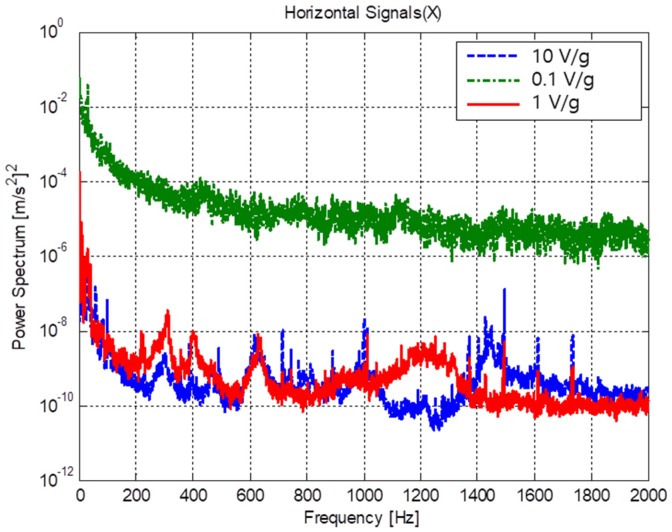
Environmental vibrations measured using accelerometers with sensitivities of 10 V/g (blue dashed line), 1 V/g (red line), and 0.1 V/g (green dash-dotted line).

## Appendix A. Selection of Accelerometer for Measurement of Environmental Vibration

In this study, sensors were selected to characterize the environmental vibrations affecting the precise equipment. Assessing the environmental vibrations for a facility in which precision equipment such as SEM or transmission electron microscope (TEM) operate requires sensors with high sensitivity and accuracy. This requirement arises from the fact that micro-or nano-level vibrations must be measured and evaluated based on stringent vibration criteria [7]. To select an appropriate sensor for measuring the environmental vibrations, three accelerometers with different sensitivities (10, 1, and 0.1 V/g) were first installed on the floor (base) of the facility. Measurements were conducted during the daytime to reflect the typical equipment operation time. The environmental vibrations were measured for a period of 100 s with Pulse (B&K, Denmark). The environment was controlled during the measurements while ensuring no personal movements or machine operations to reflect the actual operational conditions of the precise equipment.

The measured vibrations in the time domain were transformed into a power spectrum in the frequency domain to identify the frequency characteristics of the environmental vibrations because ISO 2631 provides the vibration criteria as power spectra in the frequency domain. The sampling frequency, resolution, and ensemble average were set to 1000 Hz, 0.125 Hz, and 100 cycles, respectively, following the signal processing theories [23].

Figure A1 shows the environmental vibrations measured with three different accelerometers. The blue dashed line, red line, and green dash-dotted line represent the power spectra measured using accelerometers with sensitivities of 10, 1, and 0.1 V/g, respectively. These results suggest that the accelerometer with a sensitivity of 0.1 V/g cannot provide sufficient sensitivity for the measurement of nano- to micro-level vibrations; thus, accelerometers with sensitivities of 10 V/g or 1 V/g are appropriate for these measurements. Details of the environmental vibration characteristics are described in Section 3.1.

## Appendix B. VC-Curve

**Table A1:** Vibration criteria (VC) for nano-or micro-electro-mechanical systems (MEMS) equipment [5,6,7,8] are summarized below.

Curve Criterion	Amplitude ^(1)^ μm/s (μin/s)	Detail Size ^(2)^ μm	Description of Use
Workshop (ISO)	800 (32,000)	N/A	Distinctly perceptible vibration. Appropriate for workshops and non-sensitive areas.
Office (ISO)	400 (16,000)	N/A	Perceptible vibration. Appropriate for offices and non-sensitive areas.
Residential Day (ISO)	200 (8000)	75	Barely perceptible vibration. Appropriate for sleep areas in most instances. Usually adequate for computer equipment, hospital recovery rooms, semiconductor probe test equipment, and microscopes less than 40×.
Op. Theatre (ISO)	100 (4000)	25	Vibration not perceptible. Suitable in most instances for surgical suites, microscopes to 100×, and for other equipment with low sensitivity.
VC-A	50 (2000)	8	Adequate in most instances for optical microscopes up to 400×, microbalances, optical balances, proximity, and projection aligners, etc.
VC-B	25 (1000)	3	Appropriate for inspection and lithography (including steppers) to line widths of 3 μm.
VC-C	12.5 (500)	1–3	Appropriate standard for optical microscopes up to 1000×, inspection and lithography inspection equipment (including moderately sensitive electron microscopes), to 1 μm detail size, and thin film transistor liquid crystal display (TFT-LCD) stepper/scanner processes.
VC-D	6.25 (250)	0.1–0.3	Suitable in most instances for the most demanding equipment including electron microscopes (transmission electron microscopes (TEMs) and scanning electron microscopes (SEMs)) and E-Beam systems.
VC-E	3.12 (125)	<0.1	A challenging criterion to achieve. Assumed to be adequate for the most demanding sensitive systems including long path, laser-based, and small target systems; E-Beam lithography systems working at nanometer scales; and other systems requiring extraordinary dynamic stability.

^(1)^ As measured in one-third octave bands of frequency over the frequency range of 8–80 Hz (VC-A and VC-B) or 1–80 Hz (VC-C through VC-E); ^(2)^ The detail size refers to line width in the case of microelectronics fabrication and particle (cell) size in the case of medical and pharmaceutical research, etc. It is not relevant to imaging associated with probe technologies, atomic force microscopes (AFMs), and nanotechnology.
